# Risk factors for venous thromboembolism in Slovenian children and adolescents: a single center experience

**DOI:** 10.3389/fped.2025.1729489

**Published:** 2026-01-06

**Authors:** Mineja Leban, Marko Kavčič, Jakob Peterlin, Janez Jazbec, Barbara Faganel Kotnik

**Affiliations:** 1 University Medical Center Ljubljana, Ljubljana, Slovenia; 2 Division of Pediatrics, Department of Hematooncology, University Medical Centre Ljubljana, Ljubljana, Slovenia; 3 Faculty of Medicine, University of Ljubljana, Ljubljana, Slovenia

**Keywords:** acquired risk factors, genetic thrombophilia markers, inherited thrombophilia, pediatrics, venous thromboembolism

## Abstract

Venous thromboembolism (VTE) are rare but potentially life-threatening conditions in children, usually associated with underlying medical conditions. Some children with diagnosed VTE have genetic risk factors for the development of VTE, as well as for recurrent complications. This study reports risk factors for developing VTE in a homogeneous population of children and adolescents. A total of 155 children and adolescents, aged 0–21 years, who were diagnosed with VTE at the University Children's Hospital, UMC Ljubljana, between July 2006 and October 2021, were included. The median age at the time of the VTE diagnosis was 12.0 years (interquartile range: 1–7 years). Associated medical conditions were present in 75.5% of patients, and thrombophilia was diagnosed in 43.2% of patients. Oncological disease accounted for 27.7% of cases, while infections were found to be the most significant acquired risk factor (17.4%), followed by the presence of a central venous catheter (15.5%). Genetic thrombophilia markers were identified in 27.1% of patients, with the highest frequency in adolescents (62.5%). Factor V (FV) Leiden heterozygote was the most common marker (9.6% of patients), followed by elevated factor VIII (FVIII) activity (5.8%) and elevated Lp(a) levels (5.2%). Combined thrombophilia markers were found in 52.2% of patients. In addition to inherited thrombophilia, 83.3% of patients had acquired risk factors. Compared to previously reported prevalence, a lower occurrence of FV Leiden heterozygote, elevated Lp(a) levels, elevated FVIII activity and antiphospholipid syndrome was observed in our population.

## 1 Introduction

Venous thromboembolism (VTE), encompassing deep vein thrombosis (DVT) and pulmonary embolism (PE), is rare but potentially life-threatening condition in children (1–4). Its incidence has been increasing, largely due to improvements in diagnostics and treatment of critically ill children (2, 5–7). The estimated annual incidence of VTE is estimated at 0.07–0.14 per 10,000 in the general population and at 5–8 per 10,000 among hospitalized children (6, 8–10). The highest incidence is observed in the neonates, with a second peak during adolescence, where girls are twice as likely to develop VTE compared to boys (9–13).

The mortality rate due to VTE is reported at 2.2%–8.4%, with recurrent thrombosis occurring in 6%–8% of children and post-thrombotic syndrome in 12% of children (3, 9, 10, 14).

Both acquired factors and genetic thrombophilia markers contribute to the development of VTE in children (1, 15, 16). A risk factor can be identified in approximately 80% of cases, most commonly associated with underlying conditions or known triggers (10, 17). Central venous catheter (CVC) is the most significant risk factor, reported in over 90% of neonates and in more than 50% of older children with DVT (8, 12, 15). Oncological disease is present in about 20% of affected children (18). Idiopathic VTE accounts for 2%–8.5% of cases, occurring most commonly in older children and adolescents (10).

The prevalence of inherited thrombophilia among children and adolescents with VTE varies widely (13%–79%), reflecting the clinical and demographic heterogeneity of study populations (19, 20). It is more common in older children and adolescents, particularly in cases of idiopathic VTE, where no other risk factors are identified (19, 20).

There are no unified recommendations regarding testing for inherited thrombophilia, determining the duration of anticoagulant therapy based on test results, or the role of prophylactic anticoagulation in children (10, 19). The impact of genetic thrombophilia markers on treatment duration and the potential introduction of preventive thromboprophylaxis remains unclear due to insufficient data (10, 19).

Identifying inherited thrombophilia can help determine the cause of idiopathic VTE (especially in adolescents) and identify children with high-risk prothrombotic factors, who could benefit from long-term anticoagulant therapy (15, 16, 19). Testing is also recommended for children with a positive family history or recurrent VTE (15, 21). Identified prothrombotic factors can guide lifestyle adjustments, the use of prophylaxis during temporary risk situations, and informed decisions regarding oral contraception for girls, potentially reducing the risk of recurrent VTE (19, 22).

In the present study, risk factors for VTE in a homogeneous population of children and adolescents are reported, aiming to contribute to the awareness and knowledge of this rare but important pathology.

## 2 Methods

### 2.1 Study design

A total of 155 patients, aged 0–21 years, who were diagnosed with DVT or PE at a single tertiary center, were retrospectively enrolled in the present study. All patients were treated by hematologists from the Department of Hematology and Oncology at the University Children's Hospital, UMC Ljubljana, between July 2006 and October 2021.

### 2.2 Ethics

The study was conducted in accordance with the ethical standards of the Declaration of Helsinki. The research protocol was reviewed and approved by the National Medical Ethics Committee of the Republic of Slovenia (no. 0120-85/2022/3).

### 2.3 Study population

Based on the International Classification of Diseases 10th revision (ICD-10), all children and adolescents with registered diagnosis codes for VTE (I81, I820, I822, I823, I828, I829, I260, I269, I676, I800, I801, I802, I803, I808, I809) were identified.

Using the ISPEC database, clinical data was retrospectively retrieved from medical documentation as part of regular hospital or clinical treatment. Missing data was obtained from archived medical records. Clinical data collection included patient demographics and disease characteristics, underlying clinical conditions, acquired risk factors and clinical laboratory test results [inherited thrombophilia, procoagulant blood proteins, homocysteine, Lp(a), lupus anticoagulants, anticardiolipin antibodies, antiβ2GPI].

The following acquired risk factors were assessed: family history of VTE, oral contraceptive use, infections, trauma or surgery, unhealthy lifestyle (smoking, obesity), immobilization or dehydration, presence of a CVC, chronic diseases, anatomic malformations, polycythemia, infection in combination with an inserted CVC, and oncological disease. Of the various types of *MTHFR* polymorphism, only a homozygous mutation in the *MTHFR* gene, together with homocysteinemia, was considered a risk factor for thrombophilia.

Of the total group of 232 patients, 77 were excluded from the study. Exclusion criteria included: 1) medical following because of a positive family history, 2) unproven VTE, 3) arterial thrombosis, 4) arterial cerebrovascular event, 5) parents’ testing for thrombophilia, 6) incomplete diagnostic data and 7) missing age or site of venous thrombosis. A flow diagram illustrating the selection of the study population is provided in the Supplementary Material.

### 2.4 Laboratory testing

Laboratory tests were performed during the acute phase but before treatment initiation. All participants were tested for all thrombophilias evaluated in the study. Mutations in the *MTHFR*, FV and FII genes were routinely tested in the Specialized Hematology Laboratory of Division of Internal Medicine, UMC Ljubljana, while the concentration of PC, PS, FVIII, FIX, FXII and AT were measured in the Hematology Laboratory of University Children's Hospital, UMC Ljubljana. The levels of Lp(a) and homocysteine were measured in the Biochemical Laboratory of the Institute of Clinical Chemistry and Biochemistry, UMC Ljubljana. The tests performed used units of μmol/L for homocysteine, mg/L for lipoprotein(a), and percentages for PC, PS, and AT serum levels. Allele-specific PCR with hybridization probes was employed for the genetic analysis. The selection of thrombophilia laboratory tests was based on literature available at the time, although only the following are currently considered as standardized risk factors for VTE: FV Leiden mutation, FII mutation, PC deficiency, PS deficiency, AT deficiency and antiphospholipid antibodies (APLAs) (23).

### 2.5 Statistical analysis

The selected study cohort was divided into six age groups. Descriptive statistics were used to define general and clinical characteristics. Distribution by gender was described using frequencies, and distribution by age was described using the median, interquartile range (25th–75th percentile) and age range at the time of first and recurrent VTE. Categorical variables of clinical characteristics were presented with absolute and relative frequencies.

Statistical significance differences between the frequencies of thrombophilia in our study and the prevalence reported in the literature were assessed using a binomial exact test (*p*-value, 95% confidence interval). For all hypothesis testing, a *p*-value of <0.05 was considered statistically significant. The Bonferroni-Holm method was used to adjust *p*-values. Microsoft Excel and R program (version 4.1.0.) were used for all statistical analyses.

## 3 Results

### 3.1 Clinical characteristics

The final cohort consisted of 155 patients treated for VTE. Of these, 54.2% (*n* = 84) were male and 45.8% (*n* = 71) were female. The median age at first diagnosis was 12.0 years (interquartile range: 1–7 years). The frequency of VTE was the highest among adolescents aged 16–21 years (36.1%), with a second peak during the neonatal period and a lower occurrence during childhood.

A total of 7.1% of all patients experienced recurrent VTE, with a median age of 18.0 years (range: 5–19 years). Of these, 9 were identified as having genetic thrombophilia markers, and the other 2 had an oncological condition.

### 3.2 Risk factors

Acquired risk factors or underlying diseases were found in 75.5% (*n* = 117) of patients. Oncological disease accounted for 27.7% (*n* = 43) of cases, with 51.1% of these being acute lymphoblastic leukemia. The distribution of various acquired risk factors is shown in Table 1. Bacterial infections appeared to be the most significant risk factor, while other common factors included a positive familial history, the use of oral contraceptives and the presence of a CVC. Idiopathic VTE was diagnosed in 12.9% (*n* = 20) of patients.

**Table 1:** Identified risk factors in children and adolescents with VTE.

Risk factors	*n* (%) *N* = 155
Family history of thrombophilia	22 (14.2)
Oral contraceptive pill	16 (10.3)
Infection*/inflammation	27 (17.4)
Surgery and trauma	15 (9.7)
Unhealthy lifestyle	9 (5.8)
Immobilization and dehydration	6 (3.9)
Central venous catheter	24 (15.5)
Chronic diseases	14 (9.0)
Oncological disease	43 (27.7)
Anatomic malformations	7 (4.5)
Polycythemia	4 (2.6)
Infection and central venous catheter	16 (10.3)

n/N (%), absolute frequency (relative frequency).

* Bacterial infections: sepsis, bacterial meningitis, acute mastoiditis, acute otitis media, parapharyngeal abscess, bacterial tracheitis, pneumonia with empyema, urosepsis, necrotizing enterocolitis, cellulitis.

### 3.3 Thrombophilia

**Figure 1:**
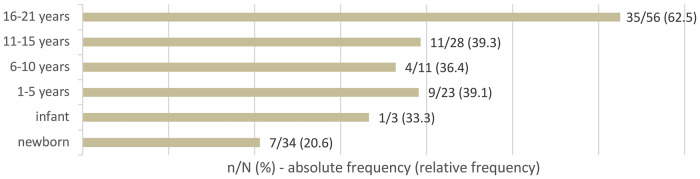
Age distribution of patients with diagnosed thrombophilia.

The overall occurrence of thrombophilia was found to be 43.2% (*n* = 67). Thrombophilia as a solitary risk factor was present in 11.9% (*n* = 8) of patients. Thrombophilia markers, either single or multiple, were most commonly identified in adolescents (62.5%). The age distribution of patients with thrombophilia is shown in Figure 1.

Genetic thrombophilia markers were found in 27.1% (*n* = 42) of patients. In addition to genetic thrombophilia markers, other acquired risk factors were found in 83.3% of patients. The distribution of identified thrombophilia markers is presented in Table 2. Factor V Leiden was the most frequently found marker, followed by elevated factor VIII activity and elevated Lp(a). Combined thrombophilia markers were diagnosed in 52.2% (*n* = 35) of patients. Antiphospholipid syndrome was the most frequent cause of acquired thrombophilia.

**Table 2:** Inherited and acquired thrombophilia markers identified in children and adolescents with VTE.

Type of thrombophilia	*n* (%) *N* = 155
*Jak2*	1 (0.6)
Elevated Lp(a)	8 (5.2)
Homocysteinemia	2 (1.3)
Antithrombin	1 (0.6)
FV Leiden homozygote	2 (1.3)
FV Leiden heterozygote	15 (9.6)
Antiphospholipid syndrome	10 (6.5)
Elevated FVIII activity	9 (5.8)
Protein C deficiency	4 (2.6)
FII heterozygote	6 (3.9)
Protein S deficiency	2 (1.3)
Low plasminogen	1 (0.6)
Acquired PC, PS, AT deficiency	3 (1.9)
Transiently positive APLAs	6 (3.9)
PC, AT, PS deficiency in oncological patients	16 (10.3)

n/N (%), absolute frequency (relative frequency).

FV, factor V; FII, prothrombin; FVIII, factor VIII; PC, protein C; PS, protein S; AT, antithrombin; Jak2, Janus kinase; APLAs, antiphospholipid antibodies.

### 3.4 Comparison to the prevalence of thrombophilia in the literature

The results of the binomial exact test for thrombophilia tests are shown in Table 3. Compared to the prevalence reported in the literature, a statistically significant (*P* < 0.05) lower incidence of the following individual thrombophilia markers was observed: a heterozygous mutation in the FV gene, elevated Lp(a) levels, antiphospholipid syndrome, and elevated activity of factor VIII.

**Table 3:** Thrombophilia markers in the study cohort compared to the prevalence reported in the literature (8, 19, 28–31, 37, 38).

Type of Thrombophilia	Study Cohort (%)	Literature (%)	Adjusted *P* Value (Confidence Interval)
Thrombophilia	43.2	13–79* ^(19)^	<0.001 (35.30–51.41)
FV Leiden heterozygote	9.6	20 ^(29)^	0.0125 (5.52–15.46)
FV Leiden homozygote	1.3	1 ^(36, 37)^	1 (0.16–4.58)
Jak2	0.6	0.88 ^(30)^	1 (0.02- 3.54)
Homocysteinemia	1.3	5 ^(29)^	0.3401 (0.16–4.58)
Elevated Lp(a)	5.2	20 ^(8)^	<0.001 (2.25–9.92)
Antiphospholipid syndrome	6.5	23.8 ^(28)^	<0.001 (3.14–11.54)
Elevated FVIII activity	5.8	25 ^(27)^	<0.001 (3.14–11.54)
FII heterozygote	3.9	3 ^(29)^	1 (1.43–8.23)
Protein C deficiency	2.6	3 ^(8, 29)^	1 (0.71–6.48)
Protein S deficiency	1.3	2 ^(8, 29)^	1 (0.16–04.58)
Antithrombin	0.6	1 ^(8, 29)^	1 (0.02–3.54)
Low plasminogen	0.6	0.5 ^(29)^	1 (0.02–3.54)

FV, factor V; FII, prothrombin; FVIII, factor VIII; Jak2, janus kinase.

* The extremes of the range values were considered.

## 4 Discussion

Thrombophilia has been established as an independent risk factor for both the development of a first VTE and recurrent VTE (24, 25).

The prevalence of thrombophilia in children and adolescents with VTE varies widely across regions due to the demographically and clinically heterogeneous pediatric populations (16, 19, 20), ranging from as low as 10% in the Canadian Registry, where a high proportion of cases involves catheter-related thrombosis in early childhood, to as high as 78% in the German Registry, which includes children of a higher median age and both arterial and cerebral thrombosis events (9, 16, 19, 26).

In our study, thrombophilia—either inherited or acquired—was identified in 43.2% of patients, and approximately half of them had combined thrombophilia markers. Considerably lower prevalence of thrombophilia was reported in the Dutch study (24). In the German study, genetic thrombophilia markers were identified in two-thirds of children with a first spontaneous VTE, and combined markers were present in one fifth of cases (25).

VTE demonstrate a bimodal age distribution (9, 10). We found neonates, infants and adolescents to be at greatest risk for VTE. Thrombophilia was most frequently identified in adolescents and was rare in neonates, which is consistent with the previous studies (6, 19, 20, 27). Higher prevalence of thrombophilia was reported in older children with idiopathic VTE, whereas lower prevalence of thrombophilia markers was observed among an unselected group of neonates and children with VTE (16). In neonates and infants, the combination of critical illness requiring intensive care and reduced coagulation activity due to an immature hemostatic system represents the predominant risk profile.

The heterozygous mutation in the FV gene, the most common single risk factor for VTE (8), was also the most frequently identified thrombophilia marker in this study. Elevated Lp(a), elevated FVIII activity and antiphospholipid syndrome were among the other commonly observed. However, significantly lower occurrences of these thrombophilia markers were observed compared to reports in the literature (8, 25, 28, 29).

The occurrence of congenital deficiencies of PC, PS, or AT, which are rarer but more serious forms of thrombophilia (29), was consistent with the literature (8, 30). In a meta-analysis of the association between genetic thrombophilia markers and VTE in children, the highest risk for the development of VTE was observed with combined thrombophilia markers and deficiencies of PC, PS, and AT (1).

In the present study, only a few patients experienced splanchnic vein thrombosis (SVT). Previous studies have shown that the *Jak2* mutation is common in patients with SVT, but not in other sites of VTE (31–33). A meta-analysis identified high prevalence and strong association between the *Jak2* mutation, SVT, and the subsequent diagnosis of myeloproliferative neoplasms (31). Based on these findings, screening for the *Jak2* mutation as part of routine thrombophilia testing seems reasonable for patients with SVT, but not for those with VTE at other sites.

Recurrent VTE was most frequently observed in adolescents, reinforcing the findings that the risk for recurrent VTE increases with age (1, 14, 25). Recurrence was more common among patients with thrombophilia, consistent with the published meta-analysis showing that most genetic thrombophilia markers—except FV mutation and elevated Lp(a)—increase the risk of recurrence (1, 34). In the German study investigating patients with an idiopathic first VTE, the risk of recurrent VTE appeared to be significantly higher in patients carrying a single or combined genetic thrombophilia marker (25). However, the presence of one or more genetic thrombophilia markers was not the predictor of recurrence in another Dutch study, likely due to the low number of idiopathic VTE cases (24).

There are no clear recommendations regarding thrombophilia testing in the children and adolescent population. Given the importance of identifying patients at increased risk of first and recurrent VTE when making treatment decisions, adolescents, as well as patients with idiopathic or recurrent VTE, are likely to benefit most from thrombophilia testing (19). Patients diagnosed with thrombophilia should also be considered for prolonged anticoagulant therapy or intermittent antithrombotic prophylaxis in situations where additional risk factors for thrombosis are present.

The majority of children and adolescents with identified thrombophilia markers also had at least one additional risk factor, consistent with previous findings (24, 27, 35). This aligns with the observation that, unlike in adults, VTE in children and adolescents typically results from a combination of inherited and acquired prothrombotic factors (1, 15, 16). We identified at least one acquired risk factor or underlying condition in three-quarters of the children, consistent with published data reporting risk factors in 67%–80% of pediatric patients (10, 17) and with a published meta-analysis showing that 70% of VTE cases include at least one clinical risk factor (1).

Consistent with previous studies, the presence of a CVC was the single most important risk factor for VTE in neonates and younger children (3, 4, 6, 9, 29). Among older children and adolescents, various additional underlying risk factors besides CVC have been reported (6). In this study, bacterial infection was the most commonly identified overall risk factor, followed by the insertion of a CVC and a positive family history. This finding underscores the potential relevance of incorporating inflammatory monitoring and infection control in VTE risk assessment. The use of OCP was considerably lower compared with that among Danish teenagers (27). The importance of a positive family history is further highlighted by an observational study from Germany, which demonstrated that family members with PC, PS and AT deficiency have a significantly increased risk of VTE (36).

Oncological diseases in childhood are an important risk factor for VTE (5). In this study, nearly one-third of the VTE cases were associated with oncological diseases, with acute lymphoblastic leukemia being the most commonly diagnosed of them, consistent with previous studies (5, 18). More than half of patients (58.1%) had at least one additional risk factor identified; predominantly the use of CVC, either alone or combined with a bacterial infection. CVC is well-established as a major risk factor for VTE in oncological patients (5). In our study, oncological disease and treatment with L-asparaginase were associated with acquired thrombophilia, including deficiencies of PC, PS, and AT. Therefore, measuring PS, PC, and AT levels in these subgroups and addressing any deficiencies appropriately may therefore be warranted.

The limitation of this study is a relatively small number of patients who met all inclusion criteria, especially when subgroups are analyzed, resulting in the limited value to detect significant associations. An additional formal statistical comparison between age groups would be appropriate to further clarify observed differences. Since a control group for comparison was not available, definitive conclusions about the exact roles of various risk factors in the development of VTE could not be drawn. Another important limitation of the study is its retrospective design. Also, because thrombophilia testing was conducted during the acute phase of thrombosis, transient reductions in PC, PS, and AT levels may be contributed to the thrombus formation itself.

The study's strengths include its single tertiary center design, which makes the cohort representative of the national children and adolescent population, although the homogeneity of the tested population may also represent a limitation. Additionally, standardized diagnostic procedures and laboratory analyses were applied uniformly to all patients.

In conclusion, our findings confirm that multiple risk factors are usually involved in in the pathogenesis of VTE in children and adolescents. However, since not all children and adolescents with these risk factors will develop VTE, it appears that a subgroup of children and adolescents with inherited thrombophilia in high-risk situations may have an increased risk for VTE. Therefore, in general, consideration should be given to the use of prophylactic anticoagulation in selected high-risk cases.

## Data Availability

The raw data supporting the conclusions of this article will be made available by the authors, without undue reservation.
