# Supplementary material for: Risk factors for venous thromboembolism in Slovenian children and adolescents: a single center experience
Source: Front Pediatr. 2026 Jan 6;13:1729489. doi: 10.3389/fped.2025.1729489 (PMC12816340; doi:10.3389/fped.2025.1729489)
Supplement: Supplementary file 1 [file Supplementaryfile1.docx]

**Flow diagram 1**: Selection of the study population

EXCLUSION #1:

- Tested due to a positive family history (n=42)

EXCLUSION #2:

- Unproven VTE (n=5)
- Arterial thrombosis (n=2)
- Arterial cerebrovascular event (n=6)

EXCLUSION #3:

- Incomplete diagnostic data (n=10)
- Missing age or site of VTE (n=12)

VTE – venous tromboembolism
